# Comparison of the Airtraq^® ^and Truview^® ^laryngoscopes to the Macintosh laryngoscope for use by Advanced Paramedics in easy and simulated difficult intubation in manikins

**DOI:** 10.1186/1471-227X-9-2

**Published:** 2009-02-13

**Authors:** Sajid Nasim, Chrisen H Maharaj, Ihsan Butt, Muhammad A Malik, John O' Donnell, Brendan D Higgins, Brian H Harte, John G Laffey

**Affiliations:** 1 Department of Anaesthesia, Galway University Hospitals, Galway, Ireland; 2 Department of Anaesthesia, Sligo General Hospital, Sligo, Ireland; 3 Department of Emergency Medicine, Galway University Hospitals, Galway, Ireland; 4 Department of Anaesthesia, Clinical Sciences Institute, National University of Ireland, Galway, Ireland

## Abstract

**Background:**

Paramedics are frequently required to perform tracheal intubation, a potentially life-saving manoeuvre in severely ill patients, in the prehospital setting. However, direct laryngoscopy is often more difficult in this environment, and failed tracheal intubation constitutes an important cause of morbidity. Novel indirect laryngoscopes, such as the Airtraq^® ^and Truview^® ^laryngoscopes may reduce this risk.

**Methods:**

We compared the efficacy of these devices to the Macintosh laryngoscope when used by 21 Paramedics proficient in direct laryngoscopy, in a randomized, controlled, manikin study. Following brief didactic instruction with the Airtraq^® ^and Truview^® ^laryngoscopes, each participant took turns performing laryngoscopy and intubation with each device, in an easy intubation scenario and following placement of a hard cervical collar, in a SimMan^® ^manikin.

**Results:**

The Airtraq^® ^reduced the number of optimization manoeuvres and reduced the potential for dental trauma when compared to the Macintosh, in both the normal and simulated difficult intubation scenarios. In contrast, the Truview^® ^increased the duration of intubation attempts, and required a greater number of optimization manoeuvres, compared to both the Macintosh and Airtraq^® ^devices.

**Conclusion:**

The Airtraq^® ^laryngoscope performed more favourably than the Macintosh and Truview^® ^devices when used by Paramedics in this manikin study. Further studies are required to extend these findings to the clinical setting.

## Background

Paramedics are frequently required to perform tracheal intubation, a potentially life-saving manoeuvre in severely ill patients, in the prehospital setting. While intubation of the trachea in the prehospital setting can be a life-saving manoeuvre [1-3], direct laryngoscopy in this setting, such as in a multiple trauma patient, is potentially difficult. Failed tracheal intubation in this context constitutes an important cause of morbidity, arising from direct airway trauma and the systemic complications of hypoxia [4,5]. In Ireland, Advanced Paramedics (AP's) are a subgroup of Emergency Medicine Technicians that are trained and certified as being competent in the skill of direct laryngoscopy and tracheal intubation. Following training on high fidelity manikins, each AP is then seconded to a hospital for clinical training in the operating suite. Each AP must perform a minimum of 10 successful tracheal intubations under the direct supervision of a senior anaesthetist. Currently, AP's perform 10–12 tracheal intubations per person per year during their clinical practice.

**Figure 1:**
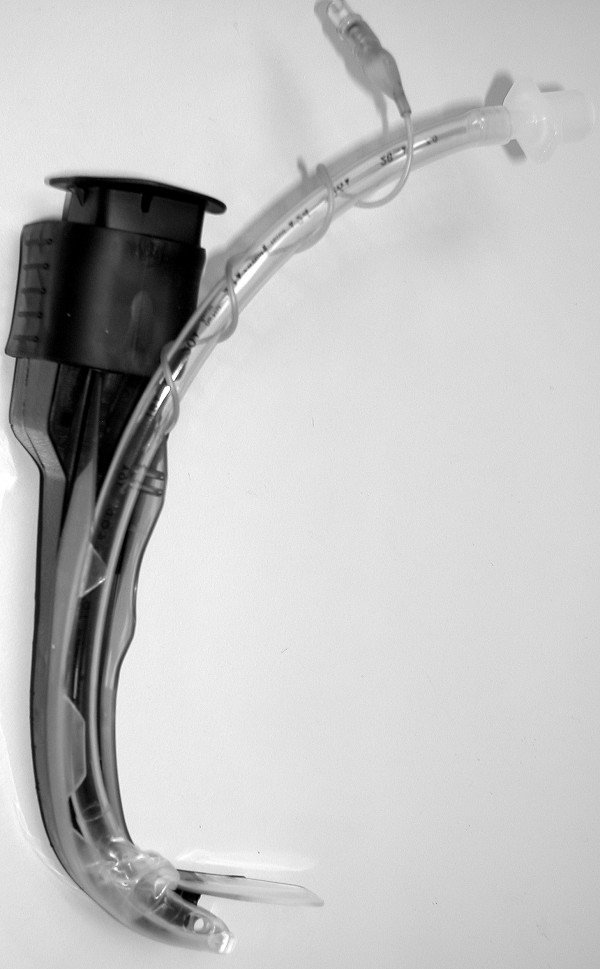
**Photograph of the Airtraq^® ^laryngoscope with a tracheal tube in place in the side channel**.

**Figure 2:**
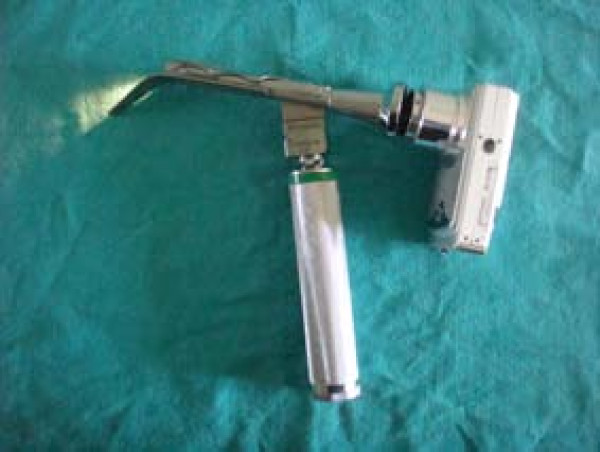
**Photograph of the Truview^® ^laryngoscope with camera attachment which clips onto eyepiece**.

The recent development of a number of indirect laryngoscopes, which do not require alignment of the oral-pharyngeal-tracheal axes, may reduce the difficult of tracheal intubation in the prehospital setting. Two relatively low cost indirect laryngoscopes, which could be easily included in ambulance equipment inventories, are the Airtraq^® ^and the Truview EVO2^® ^devices. The Airtraq^® ^device, which incorporates a side channel (Figure 1), has been demonstrated to have advantages over the Macintosh have when used by both paramedic students and experienced paramedics [6]. The Truview EVO2^® ^(Truphatek International Ltd, Netanya, Israel) laryngoscope blade [7] (Figure 2), which is essentially a modification of the Macintosh blade, may require less adjustment in laryngoscopy technique from that used with the Macintosh. The efficacy of the Truview^® ^when used by paramedics is not known, and the relative efficacies of these devices in comparison to the Macintosh have not been compared in a single study. We therefore wished to compare the relative efficacies of these devices, and their efficacy compared to the Macintosh laryngoscope when used by paramedics with demonstrated competence in the skill of tracheal intubation using the Macintosh laryngoscope.

## Methods

Following ethical committee approval, and written informed consent, 21 Advanced Paramedics certified as competent to perform tracheal intubation consented to participate in this study. These participants constituted a convenience sample of AP's that attended a Resuscitation Conference on the 7 – 9^th ^December 2007 in Limerick, Ireland, and represents more than 20% of all paramedics in Ireland.

Each AP received a standardized training session with the Airtraq^®^, the Truview^® ^and the Macintosh laryngoscopes. This included a demonstration of the intubation technique with each device, and verbal instructions regarding the correct use of each device. The use of optimization manoeuvres, such as external laryngeal pressure, to facilitate intubation with the Macintosh was also demonstrated. The total training time for each device was five minutes. Each participant was then allowed to perform practice attempts with each device until each performed one successful tracheal intubation with each device. This training was carried out by a different member of the study team to the investigator that performed the actual study measurements. All intubations were performed with a 7.5 mm internal diameter cuffed endotracheal tube (ETT). We used the Truview EVO2^® ^with its camera attachment on the top of blade (Figure 2) in order to magnify the view from the eyepiece. The sequence in which each participant used the devices was initially randomized, and thereafter each participant used the devices in the same sequence throughout the protocol.

The design of the study was a randomized crossover trial. Each AP performed tracheal intubation with each device in a SimMan^® ^manikin (Laerdal^®^, Kent, UK) in the following laryngoscopy scenarios: (1) normal airway in the supine position; and (2) following the application of a hard neck collar. A maximum of 3 intubation attempts were permitted with each device in each scenario. The primary endpoint was the duration of successful tracheal intubation attempts. The duration of each tracheal intubation attempt was defined as the time taken from insertion of the blade between the teeth until the ETT was deemed to be correctly positioned by each participant. Endotracheal tube placement was determined by each participant by direct visualization. Where the participant was unsure as to the position of the ETT, the time taken to connect the ETT to an Ambu^® ^bag and inflate the lungs was also included in the duration of the attempt. In any case, after each intubation attempt an investigator verified the position of the ETT tip. A failed intubation attempt was defined as an attempt in which the trachea was not intubated, or where intubation of the trachea required greater than 60 seconds to perform.

Additional endpoints included the rate of successful placement of the endotracheal tube (ETT) in the trachea, the number of intubation attempts, the number of optimization manoeuvres required (readjustment of head position, second assistant) to aid tracheal intubation and the severity of dental trauma. The severity of dental trauma was calculated based on a grading of pressure on the teeth (none = 0, mild = 1, moderate/severe = 2). To improve reliability the same investigator assessed the severity of dental compression every time thus removing the potential for any inter-rater variability. We have demonstrated in previous studies that this method of assessing dental pressure performs well, and appears to yield reasonably consistent results over time [8,9].

At the end of each scenario, each participant scored the ease of use of each device on a visual analogue scale (from 0 = Extremely Easy to 10 = Extremely Difficult). At the end of this protocol, each participant completed a questionnaire to determine self-assessed comfort and skill level for all three devices.

### Statistical analysis

We based our sample size estimation on the duration of the successful tracheal intubation attempt. Based on prior studies [8] we projected that the duration of tracheal intubation would be 15 seconds for the Macintosh laryngoscope, with a standard deviation of 5 seconds, in the easy laryngoscopy scenario with the Macintosh laryngoscope. We considered that an important change in the duration of tracheal intubation would be a 33% absolute change, i.e. an increase to 20 seconds or a reduction to 10 seconds. Based on these figures, using an α = 0.05 and β = 0.2, for an experimental design examining three devices, we estimated that 17 AP's would be required. We therefore aimed to enrol a minimum of 20 AP's to the study.

The analysis was performed using Sigmastat 3.5 (Systat Software, San Jose, CA, USA. Data for the duration of the first and the successful intubation attempt, the instrument difficulty score, and the overall device assessment were analyzed using one way Analysis of Variance (ANOVA) or the using the Kruskal-Wallis One Way ANOVA on Ranks depending on the data distribution. Data for the number of intubation attempts, number of optimization manoeuvres, severity of dental trauma, and the instrument difficulty score were analyzed using the Kruskal-Wallis One Way Analysis of Variance on Ranks. Data for the success of tracheal intubation attempts was analyzed using Chi square or Fishers exact test as appropriate. Continuous data are presented as means ± standard deviation (SD) or median (interquartile range), and ordinal and categorical data are presented as number and as frequencies. The α level for all analyses was set as P < 0.05.

## Results

Twenty-one Advanced Paramedics were approached and each consented to participate, and were enrolled into the study.

### Scenario 1 – Normal airway scenario

**Figure 3:**
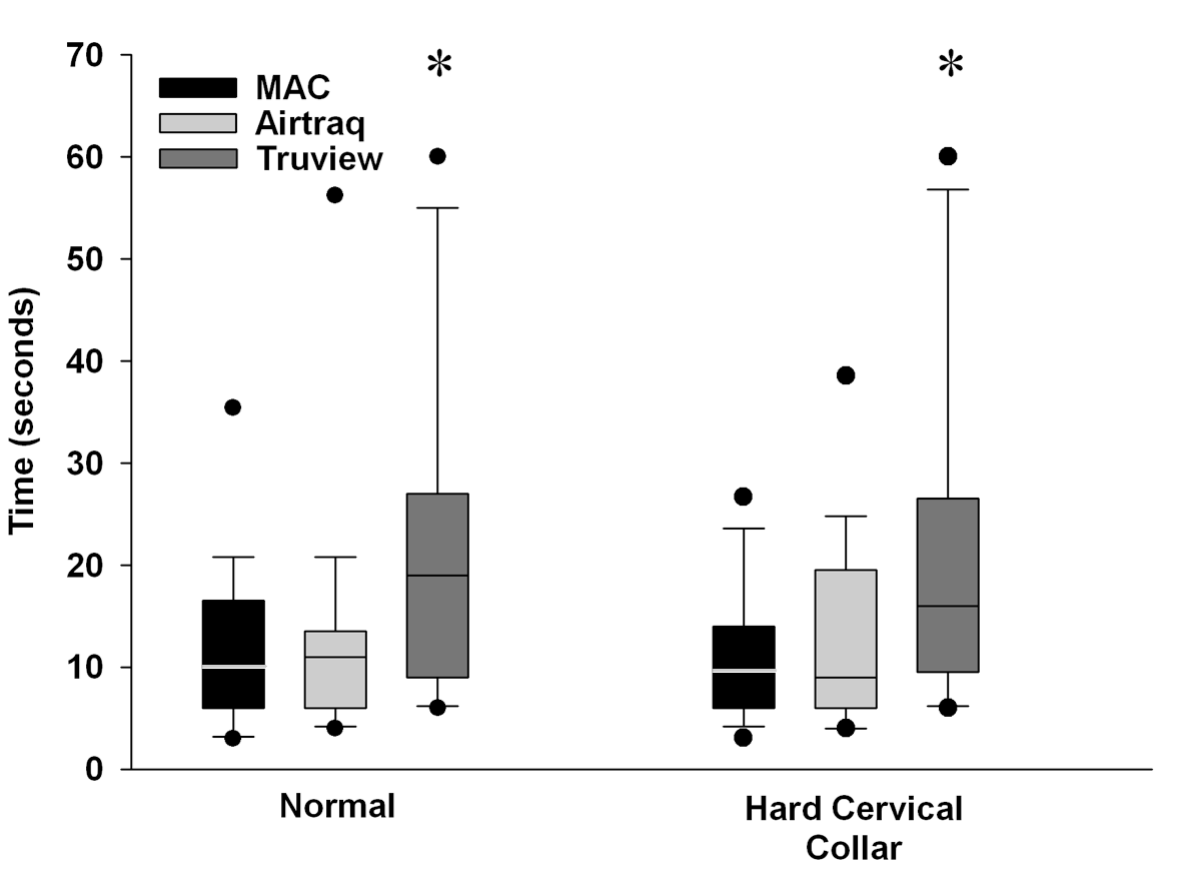
**Box plot representing the duration required to successfully intubate the trachea with each device in each scenario tested**. The data are given as median and interquartile range, with the bars representing the 10^th ^and 90^th ^centile. * Indicates significantly different compared to both other Laryngoscopes. Labels: Normal: Intubation of the normal airway; Cervical Immobilization – Immobilization of the neck with hard collar.

The duration of the first and of the successful tracheal intubation attempts were significantly greater with the Truview^® ^laryngoscope compared to the Macintosh and Airtraq^® ^laryngoscopes. There were no significant differences in the duration of the first or the successful tracheal intubation attempts between the Macintosh and Airtraq^® ^devices (Table 1 and Figure 3). All 21 AP's successfully intubated the trachea with the Macintosh laryngoscope, compared to 20 with the Airtraq^® ^and 19 with the Truview^® ^laryngoscopes (Table 1).

**Table 1:** Data from the easy laryngoscopy scenario

	**Macintosh**	**Airtraq^®^**	**TruView^®^**	**P value**
Overall Success Rate (%)	21/21 (100)	20/21 (95)	19/21 (90.5)	P = 0.597
Duration of 1^st ^intubation	9 (6, 16)	11 (6, 14)	17 (11, 60)*	P = 0.004
attempt in seconds (median, IQR) [Range]	[3 to 60]	[4 to 60]	[6 to 60]	
				
Number of Intubation Attempts (%)				
1	20 (95.3)	19 (90.5)	14 (66.7)	P = 0.063
2	1 (4.7)	1 (4.75)	3 (14.3)	
3	0	1 (4.75)	4 (19)	
				
No of Optimisation Manoeuvres (%)				
0	10 (47.6)	19 (90.5)*	0*	P < 0.001
1	11 (52.4)	2 (9.5)	14 (66.7)	
>1	0	0	7 (33.3)	
				
Severity of Dental Compression (%)				
0	0*	7 (33.3)	3 (14.3)	P < 0.001
Mild [+]	10 (47.6)	12 (57.2)	14 (66.7)	
Severe [++]	11 (52.4)	2 (9.5)	4 (19)	

**Notes: **Data are reported as median (interquartile range) [range], or as number (percentage). P value indicates the value for the initial 3 group analysis using Chi square, one way ANOVA or ANOVA on ranks (Kruskall-Wallis) as appropriate.

* Significantly [P < 0.05] different on post hoc Student-Newman-Keuls testing compared to both other devices

**Figure 4:**
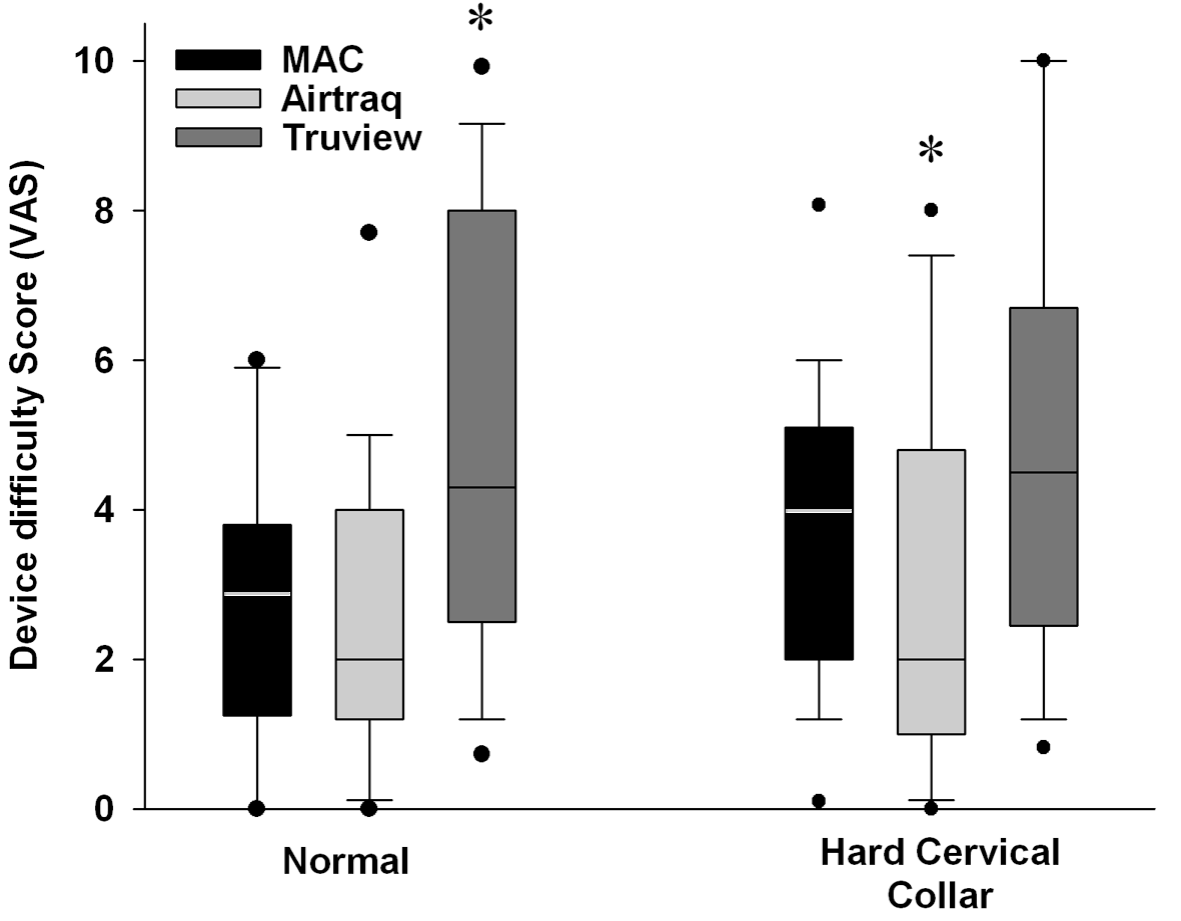
**Box Plot representing the user rated degree of difficulty of use of each instrument in each scenario tested**. The data are given as median and interquartile range, with the bars representing the 10^th ^and 90^th ^centile. * Indicates significantly different compared to both other Laryngoscopes. Labels: Normal: Intubation of the normal airway; Cervical Immobilization – Immobilization of the neck with hard collar.

There were no between group differences in the number of intubation attempts required with each device. The number of optimization manoeuvres required was significantly lower with the Airtraq^® ^group, and significantly greater with the Truview^® ^group, compared to the Macintosh group. The severity of dental compression was significantly greater with the Macintosh group compared to both the Airtraq^® ^and Truview^® ^devices (Table 1). The participants found the Truview^® ^significantly more difficult to use than the other laryngoscopes in this scenario. There was no significant difference in the difficulty of device use between the Macintosh and Airtraq^® ^devices (Figure 4).

### Scenario 2 – Manikin with cervical collar

The duration of the successful, but not the first, tracheal intubation attempt was significantly longer with the Truview^® ^compared to the Macintosh and Airtraq^® ^laryngoscopes. There was no significant difference in the duration of these attempts between the Macintosh and Airtraq^® ^devices (Table 2 and Figure 3). All AP's successfully intubated the trachea with the Macintosh and Airtraq^® ^laryngoscopes, compared to 20 with the Truview^® ^laryngoscope (Table 2).

**Table 2:** Data from the cervical immobilization scenario

	**Macintosh**	**Airtraq^®^**	**TruView^®^**	**P value**
Overall Success Rate (%)	21/21 (100)	21/21 (100)	20/21 (95)	P = 0.362
				
Duration of 1^st ^intubation	10 (7, 14)	9 (6, 20)	17 (11, 48)	P = 0.058
attempt in seconds (median, IQR) [Range]	[3 to 60]	[4 to 60]	[6 to 60]	
				
Number of Intubation Attempts (%)				
1	18 (85.7)	18 (85.7)	16 (76.2)	P = 0.562
2	3 (14.3)	2 (9.5)	2 (9.5)	
3	0	1 (4.8)	3 (14.3)	
				
No of Optimisation Manoeuvres				
0	9 (42.9)	15 (71.4)*	0*	P < 0.001
1	9 (42.9)	6 (28.6)	17 (81)	
>1	3 (14.2)	0	4 (19)	
				
Severity of Dental Compression (%)				
0	0	11 (52.3)*	0	P < 0.001
Mild [+]	10 (47.6)	9 (42.9)	12 (57.1)	
Severe [++]	11 (52.4)	1 (4.8)	9 (42.9)	

**Notes: **Data are reported as median (interquartile range) [range], or as number (percentage). P value indicates the value for the initial 3 group analysis using Chi square, one way ANOVA or ANOVA on ranks (Kruskall-Wallis) as appropriate.

* Significantly [P < 0.05] different on post hoc Student-Newman-Keuls testing compared to both other devices

There were no between group differences in the number of intubation attempts required with each device. The number of optimization manoeuvres required was significantly lower with the Airtraq^® ^group, and significantly greater with the Truview^® ^group, compared to the Macintosh group. The severity of dental compression was significantly lower with the Airtraq^® ^compared to the Macintosh and Truview^® ^laryngoscopes. There was no difference in the severity of dental compression between the Macintosh and Truview^® ^devices (Table 2). The participants found the Airtraq^® ^significantly less difficult to use than the other laryngoscopes in this scenario (Figure 4). There was no significant difference in the difficulty of device use between the Truview^® ^and Macintosh devices.

### End Protocol overall device assessment

The AP's found the Macintosh and Airtraq^® ^laryngoscopes significantly easier to use than the Truview^® ^laryngoscope (Table 3). There was no significant difference in the ease of use of the Macintosh and Airtraq^® ^laryngoscopes. The AP's had significantly less confidence with the Truview^® ^compared to the Macintosh and Airtraq^® ^laryngoscopes. There was no significant difference in confidence with the Macintosh and Airtraq^® ^laryngoscopes (Table 3).

**Table 3:** Overall device assessment by participants

	**Macintosh**	**Airtraq^®^**	**TruView^®^**
Overall ease of Use Score	2.6 ± 1.3	2.6 ± 1.2	4.3 ± 3.0*
Overall Confidence with each device	8.4 ± 1.1	7.6 ± 1.9	5.5 ± 2.1*

**Notes: **Data are reported as mean ± SD or as number (percentage).

* Significantly [P < 0.05] different compared to both other devices

## Discussion

Several studies have demonstrated improved outcome in severely ill and injured patients if the airway is successfully secured early by tracheal intubation [1-3]. Conversely, the occurrence of difficulties and/or failure to successfully intubate the trachea constitutes an important cause of morbidity in the pre-hospital setting [4,5,10]. Tracheal intubation is frequently difficult to perform and associated with a lower success rate in this challenging environment [11]. The need for repeated attempts to secure the airway emergently increases airway-related complications such as hypoxia, pulmonary aspiration and adverse haemodynamic events [5]. Of particular concern, accidental oesophageal intubation in emergency situations outside the operating room results in high incidences of severe hypoxaemia, regurgitation and pulmonary aspiration of gastric contents, cardiac dysrythmias and cardiac arrest [4]. Difficulties in tracheal intubation may also result in severe local complications such as perforation of laryngeal or pharyngeal structures [12].

These difficulties have led several commentators to question the practice of pre-hospital tracheal intubation by personnel not fluent in the technique [13-15]. A slow learning curve for intubation with the Macintosh blade has been well documented among paramedic personnel [16,17] due to lack of regular exposure to the technique. These difficulties have led to the increasing use of supraglottic devices (Combitube^®^, Laryngeal Tube^® ^and Laryngeal Mask Airway^®^) for airway management in these contexts [18-20], due to the rapid learning curves associated with these devices [21,22]. However trauma to the airway and/or aspiration injury remains a significant risk with these devices in these patients.

Conventional direct laryngoscopes, such as the Macintosh laryngoscope, require the alignment of oral and tracheal axes in order to view the glottic opening. This is a difficult skill to successfully acquire [16,15,23], and to maintain [17], particularly if the opportunities to practice this skill are limited. The Airtraq^® ^device is an indirect laryngoscope with an exaggerated curvature with enhanced optics (Figure 1). The Truview^® ^laryngoscope (Figure 2) is essentially a modified Macintosh blade with an exaggerated distal curvature and a viewing lens that can be attached to a camera to magnify the view of the vocal cords. Both devices give a view of the glottis without the need to align the oral and tracheal axes, and therefore may simplify tracheal intubation. Both devices are relatively low cost, and could be easily included in ambulance equipment inventories. We therefore wished to compare the relative efficacies of these devices, and their efficacy compared to the Macintosh laryngoscope when used by paramedics in the setting of normal and simulated difficult tracheal intubation.

Our study demonstrated that the Airtraq^® ^demonstrated advantages over the Macintosh laryngoscope, in both the normal and in the difficult tracheal intubation scenario. The Airtraq^® ^reduced the number of optimization manoeuvres and reduced the potential for dental trauma when compared to the Macintosh laryngoscope. We did not find any difference in tracheal intubation success rates with the Airtraq^® ^device in comparison to the Macintosh in the difficult laryngoscopy scenario. This is due to the relatively high tracheal intubation success rates with all devices in our difficult airway scenario, in our study. This latter finding contrasts with that reported for experienced prehospital laryngoscopists by Woollard et al [6], who reported greater success rates with the Airtraq^® ^compared to the Macintosh. Differences between the models of difficult intubation in the two studies are likely to explain these divergent findings.

In contrast, the Truview^® ^laryngoscope did not demonstrate advantages over the Macintosh laryngoscope. In several respects it performed more poorly than the Macintosh. Of importance, the duration of intubation attempts were significantly longer with the Truview^® ^and the number of optimization manoeuvres required was greater, compared to both the Macintosh and Airtraq^® ^devices. The AP's rated this device least favourably.

Of interest, in their overall device assessment, the AP's rated the Macintosh and the Airtraq^® ^laryngoscopes similarly, notwithstanding the advantages demonstrated for the Airtraq^® ^laryngoscopes in the study. These ratings probably reflect the familiarity of the AP's with the Macintosh laryngoscope and the ease of use of the Airtraq^® ^device. Previous studies with the Airtraq^® ^have consistently demonstrated a requirement for less operator skill to use this device compared to the Macintosh laryngoscope, leading to more rapidly acquired proficiency [24-26]. The demonstration that the Airtraq^® ^exhibits a rapid learning curve, despite a deliberately brief instruction period, supports this contention and this probably accounts for its favourable rating by the AP's.

A number of important limitations exist regarding this study. First, this is a manikin study, and these findings need to be confirmed and extended in clinical studies before definitive conclusions can be drawn. Nevertheless, our findings regarding the Airtraq^® ^in manikin studies in other settings [8] have been confirmed in subsequent clinical studies [27,28], underlining the importance of the findings of this study. Second, we acknowledge that the potential for bias exists, as it is impossible to blind the AP's to the device being used. Third, this study was carried out in experienced users of the Macintosh laryngoscope. The findings may differ in studies of paramedics prior to their attaining competence with the Macintosh device. In this regard, a group of prehospital providers that had no previous training in performing tracheal intubation demonstrated high levels of success with the Airtraq^® ^[29]. In a separate study from this same group of investigators, a group of third year paramedic students and a group of experienced prehospital laryngoscopists each had increased first-time intubation rates and lower rates of oesophageal intubation with the Airtraq^® ^compared with the Macintosh laryngoscope, in a manikin model of difficult tracheal intubation [6]. Fourth, we defined a maximal permissible duration of tracheal intubation attempts of 60 seconds. A 30 second breath-to-breath interval is widely considered to be the maximum permissible duration of a tracheal intubation attempt in the pre-hospital setting. Fifth, although the study is adequately powered to detect the primary outcome, namely differences in the duration of tracheal intubation attempts, the sample size is relatively small and may therefore be subject to bias, and may not have been sufficient to detect secondary outcomes. Finally, the relative efficacies of these devices in comparison to other promising devices such as the Glidescope^® ^[30], McCoy^® ^[31], McGrath^® ^[32] or Bonfils^® ^[33] have not been determined. We focussed on the Airtraq^® ^and Truview^® ^in this study due to the fact that these are relatively low cost, portable devices that can easily be included in the equipment used by AP's. Nevertheless, further comparative studies are needed with other alternative laryngoscopy devices in order to find the optimal alternatives to the Macintosh laryngoscope.

## Conclusion

We conclude that the Airtraq^® ^laryngoscope may possess certain advantages over the conventional Macintosh laryngoscope when used by Advanced Paramedics in normal and simulated difficult intubation scenarios. The Airtraq^® ^laryngoscope constitutes a promising alternative device to the Macintosh for use by AP's. In contrast, the Truview^® ^performed less well, and does not demonstrate promise in this context. Further studies, which evaluate the efficacies of these devices in the clinical setting, are required.

## Abbreviations

ANOVA: analysis of variance; AP: advanced paramedic; ETT: endotracheal tube; SD: standard deviation; VAS: Visual analogue scale.

## Competing interests

The authors have no competing interests in regard to the Airtraq^® ^or Truview^® ^devices.

## Authors' contributions

SN and CM conceived of the study, and participated in its design and execution and helped to draft the manuscript. IB, JO'D, BDH and BH participated in the study, recruited patients, and helped to draft the manuscript. JL participated in the design and coordination of the study, performed the statistical analysis, and helped to draft the manuscript. All authors read and approved the final manuscript.

## Pre-publication history

The pre-publication history for this paper can be accessed here:


